# Epidemiology, biology, pathogenesis, clinical manifestations, and diagnosis of dengue virus infection, and its trend in Ethiopia: a comprehensive literature review

**DOI:** 10.1186/s41182-023-00504-0

**Published:** 2023-02-24

**Authors:** Biruk Zerfu, Tesfu Kassa, Mengistu Legesse

**Affiliations:** 1 Department of Medical Laboratory Science, College of Health Science, Addis Ababa University, Addis Ababa, Ethiopia; 2 Aklilu Lema Institute of Pathobiology, Addis Ababa University, Addis Ababa, Ethiopia

**Keywords:** Dengue, Biology, Epidemiology, Pathogenesis, Clinical manifestation, Diagnosis

## Abstract

Dengue fever is a dengue virus infection, emerging rapidly and posing public health threat worldwide, primarily in tropical and subtropical countries. Nearly half of the world's population is now at risk of contracting the dengue virus, including new countries with no previous history-like Ethiopia. However, little is known about the epidemiology and impact of the disease in different countries. This is especially true in countries, where cases have recently begun to be reported. This review aims to summarize epidemiology, biology, pathogenesis, clinical manifestations, and diagnosis of dengue virus infection and its trend in Ethiopia. It may help countries, where dengue fever is not yet on the public health list-like Ethiopia to alert healthcare workers to consider the disease for diagnosis and treatment. The review retrieved and incorporated 139 published and organizational reports showing approximately 390 million new infections. About 100 million of these infections develop the clinical features of dengue, and thousands of people die annually from severe dengue fever in 129 countries. It is caused by being bitten by a dengue virus-infected female mosquito, primarily *Aedes aegypti* and, lesser, *Ae. albopictus*. Dengue virus is a member of the *Flavivirus* genus of the *Flaviviridae* family and has four independent but antigen-related single-stranded positive-sense RNA virus serotypes. The infection is usually asymptomatic but causes illnesses ranging from mild febrile illness to fatal dengue hemorrhagic fever or shock syndrome. Diagnosis can be by detecting the virus genome using nucleic acids amplification tests or testing NS1 antigen and/or anti-dengue antibodies from serum, plasma, circulating blood cells, or other tissues. Dengue cases and outbreaks have increased in recent decades, with a significant public health impact. Ethiopia has had nearly annual outbreaks since 2013, devastating an already fragmented health system and economy. Standardization of medication, population-level screening for early diagnosis and prompt treatment, and minimization of mosquito bites reduce overall infection and mortality rates.

## Introduction

Dengue fever (DF) is a rapidly emerging acute febrile disease, with potentially fatal complications, of public health concern worldwide, mainly in tropical and subtropical regions [1, 2]. The term ‘dengue fever’ was derived from the Swahili word ‘Ka-dinga pepo’ synonymous with ‘cramp-like seizure’. In 1787, Benjamin Rush coined the term as “break-bone fever” because of the symptoms of myalgia and arthralgia involvement during epidemic reports in Philadelphia in 1780 [3]. DF epidemics were first recognized clinically in the 1780s in Asia, Africa, and North America at about the same time [4]. Dengue virus (DENV) that is known to cause the full range of DF diseases worldwide comprises four independent but antigen-related serotypes (DENV1-4) and belongs to the genus *flavivirus* of the family *Flaviviridae* [5]. The virus is a positive sense single-stranded encapsulated RNA virus and comprises virus-encoded three structural proteins, namely, nucleocapsid or core (C) protein, membrane-associated (M) protein and envelop (E) glycoprotein and seven non-structural (NS) proteins [3]. It is transmitted to humans by the bite of infected female *Aedes* mosquitoes [5, 6].

The DF disease ranges from mild to undifferentiated fever illness to severe dengue hemorrhagic fever (DHF) and dengue shock syndrome (DSS) [7, 8]. For management of cases, in 2009 WHO revised and classified DF disease into uncomplicated DF and severe DF, though the 1997 WHO classification that classified DF into undifferentiated fever, DF and DHF is still in use [8, 9]. The main clinical manifestations of each categories would be continuous high fever lasting 2–7 days, haemorrhage—manifested by petechiae, epistaxis, positive tourniquet test or thrombocytopenia, and plasma leakage shocks—manifested by hemoconcentration (hematocrit above 20%), pleural effusion and ascites [3]. In regions where DF case is found as endemic and/or epidemic, particularly in tropical and subtropical countries including in Ethiopia, patients with febrile illness compliance are main public disease, commonly sought medical attention [10]. In Ethiopia and in Africa as a whole, these febrile illnesses would likely caused by various infectious pathogens, complicating control and response programs to epidemic and pandemic diseases, such as malaria, Ebola and COVID-19 [11]. The manifestations of the febrile illnesses are the manifestations of DF and other arboviruses illness [12–14]. However, DF and other arboviral diseases are unknown or low, probably due to misdiagnosed or undiagnosed febrile patients [13]. In East African countries, even though few data were available so far, DF is notably recognized as epidemic-prone public health threat disease [15]. In countries where DF and other arbovirus diseases case potentially exist, accurate and multiplex diagnoses need to be employed for patient care and epidemiological surveillance for epidemic preparedness and response within the countries and at the regions at large [16].

The existing surveillance system is inadequate to detect new cases and the dynamics of the virus in a timely manner because of inadequate human, financial, material and technical resources to equip laboratory services and epidemiological capacity. Similarly, the diagnosis, diagnostic methods and health service systems variability of countries hinder the reliable estimations of burden and distributions of emerging diseases like DF disease [17]. These are the reasons why the 2015 United Nations plan through its sustainable development goals (SDGs) to end emerging or re-emerging neglected tropical diseases epidemics by 2030, which encompasses Arboviral diseases including DF, seems far from the target achievement. In Ethiopia, various DF outbreaks have occurred every year among undifferentiated febrile patients in different areas of the country, including in Afar region, in Somali region, and in Dire Dewa city administration [18–20]. Since 2013 during the first identified outbreak [21]. In addition, a serological survey has identified DF disease, revealing DF is spreading and emerging disease in the country [22]. However, the country has been hampered by challenges of lack of clinical experience to suspect and manage potential DF cases and by the absence of adequate laboratory diagnostic capabilities. As a result, DF cases possibly remain undiagnosed as mild or asymptomatic, misdiagnosed with other clinical manifestations resembling malaria, bacterial, or other viral infections or remaining unidentified febrile illness [13, 23]. Therefore, DF cases, including epidemics, emerge and pose a significant unforeseen and none-responded challenge to the country due to weak health system, low experience of health service providers, and economic instability [24]. State-of-the-art reviews tend to address current status of DENV and DF dynamics reflection would be crucial for consideration of the emerging DF cases for diagnosis and management in countries like Ethiopia, where DF cases have not yet considered as a public health important disease. This review summarizes the current knowledge of epidemiology, biology, pathogenesis, clinical manifestations and diagnosis of dengue virus infection and its trend in Ethiopia.

### Epidemiology of DENV infection

The DF was first identified and named in 1789 by Benjamin Rush, who coined the term “break-bone fever” because of the symptoms of myalgia and arthralgia. DF epidemics were occurred and recognized simultaneously in Asia, Africa, and North America in the 1780s [4]. The disease is endemic in many countries in WHO regions of Africa, the Americas, the Eastern Mediterranean, Asia, Australia and the Western Pacific [1, 25, 26], though the Americas, South–East Asia and Western Pacific regions are the most seriously affected, with Asia representing ~ 70% of the global burden of DF disease [5]. The lack of a unified and coordinated effort at the regional levels to initiate population-based epidemiological surveillance with clear operational goals leads to differences in burden reports within regions [27]. Since 1940, the risk of contracting the DENV infection has increased by over 30-fold and has widespread dramatically as population movements during World War II spread around the world [4]. In the late 1990s, DF was the second most important mosquito-borne disease after malaria, with approximately 40 million DF cases and hundreds of thousands of DHF cases each year [4]. Currently, a half of the world populations are at risk of contracting DF disease. Annually, an estimated 390 million DF infections, of which about 100 million manifest clinical features and nearly a thousand cases develop fatal DHF/DSS, predominantly in the tropical and subtropical regions including the Americas, Asia, Australia, and Africa [1, 7, 28]. The spreading and distribution of DF cases are attributed to the factors such as globalization, population and urbanization growth, variations in climate and environmental factors, lack of sanitary services, ineffective mosquito control, and increasing DENV surveillances and case reports. The factors may contribute to the increases mosquito populations and susceptibility to circulating serotypes and creating favourable temperature, precipitation, and humidity for the reproduction and feeding patterns of the mosquito populations and for the DENV incubation period [5]. Besides, in recent years, the losses of enzootic amplification requirements and adaptation for replication at higher temperatures of the vector have made DENV cause the largest and the most extensive epidemic in tropical urban areas [29]. Endemic and epidemic DF transmission cycles are augmented with significant morbidity, mortality, and economic costs, particularly in developing countries [2].

Since 2000, a sharp rise in DF incidence, the spread of cases to new countries, and the urban-to-rural spread risk of about half of the world’s population [5, 17]. The DENV's limit of infectivity has now reached 129 countries with good evidence of DF cases and outbreaks, including 36 countries previously classified as dengue-free by WHO and/or the US CDC [17]. Annually, an estimated 390 million DENV infections could have occurred, from that estimated 67–136 million cases can manifest clinical features and thousands of case develops into severe/deaths most frequently in the population of countries in tropical and subtropical regions of the world. The tendency of increase may be due to globalization, the growth of the population and urbanization, variations in climate and environmental factors, the lack of sanitary services, ineffective mosquito control and increasing DENV surveillances and case reports [5]

Over the past 20 years, the number of DF cases and deaths reported to WHO has increased by more than eightfold and fourfold, respectively. The reported cases increased from 505,430 in 2000 to more than 2.4 million in 2010 and to more than 5.2 million cases in 2019, where the reported deaths increased from 960 in 2000 to 4032 in 2015 [5]. Moreover, the cases of DF have shifted from mostly affecting children 40–50 years ago to affect all age groups [26]. During the 2020–2021 years, the total number of reported cases and deaths contrarily seemed to decrease. However, the data were not completed yet as well as the COVID-19 pandemic that hampered case reporting in several countries [5]. Even though the DF case and deaths reports have been growing over wide geographical locations and in all ages of populations, the current global distribution remains highly uncertain [17].

In Africa, the epidemiology of DF is poorly characterized, even though the vector mosquitoes present a high burden in the neighbouring Middle East and in Sub-Saharan Africa as well all serotypes of DENV circulate in 19 countries of the continent [4]. In the region of Sub-Saharan Africa, DENV infection seems a significant burden for public health, with an estimated burden of about 25% (21–29%) by IgG, 10% (9–11%) by IgM and 14% (12–16%) by viral RNA tests [30]. Moreover, many countries of the region, including Burkina Faso in 2016 and 2017, Côte d’Ivoire in 2017, Cape Verde in 2009, and Egypt in 2015 have experienced of DF outbreaks that were reported to Africa CDC [31].

### DENV infection in Ethiopia

DF is understudied in Ethiopia, but the country has a sharply increased number of DF outbreak cases, continued transmission, and increased viral infection [32]. These would be reasons to recommend Ethiopia has high potential risk factors for DENV transmission and the following findings would further substantiate the recommendation. First; *Ae. Aegypti*, the vector transmitting DENV, have been extraordinarily identified at indoor and outdoor levels [33]. From immature stages collected from discarded containers and other artificial water containers found around houses and peri-domestic areas, about 50–84% of them were morphologically identified as *Ae. aegypti* [34, 35]. Second, DF has been potentially misdiagnosed in Africa including in Ethiopia as malaria, because over-diagnosis of malaria in areas of low transmission has been well documented and overestimating of malaria (≈ 75%) by clinical diagnosis [36]. Reporting of viruses that are similar to DENV by having same transmitting vector and being in the same member of genus *Flavivirus* was started in Ethiopia during the yellow fever (YF) outbreak in 1960–62 in southern Ethiopia [37]. *Flavivirus* including Zika virus (ZIKV), West Nile virus (WNV), Chikungunya virus (CHIKV), Wesselsbron, Talaguine and Sindbis viruses were serologically identified in human populations and in wild animals in Southern and Western Ethiopia [38]. Despite potential serological cross-reactivity due to the broad antigenic cross-reactivity of antibodies and clinical presentation similarities with causing febrile illness to these *Flavivirus* infections [39], DENV infection was not identified and reported until the last decade in Ethiopia.

In Ethiopia, DENV infection outbreak was first reported in 2013 during DF related outbreak that occurred in Dire Dawa city administration from eastern part of the country. During the outbreak, 12,000 DF-related suspected cases were registered, from which 88 of the cases were confirmed by ELISA and RT-PCR and 50 of the cases were found positive for DENV infection, specifically for DENV-2 serotype [21, 33]. The next year, other many outbreaks were identified in a year round fashion in Dire Dawa city administration, in Godey Town of the Somali Region, and in the Adar district of the Afar region [18, 19]. In the Somali region, during the same months of three series of years, which were in January, February and March of 2014, 2015 and 2016, DF-related outbreaks with a total of 440 cases occurred [40]. In May 2017, DF-related outbreak from Kabridahar Town in the Somali region was reported with a total of 101 cases, including five with severe DF and one death [19]. Similarly, the epidemiological evidence reveals that DF-related outbreaks were recorded from 2017 to 2021 in the Somali region and Dire Dewa city administration [19, 41–43]. Furthermore, from Jan 01 to Feb 04, 2021, Ethiopian health officials reported DF-related outbreaks of 160 confirmed DENV infection cases in Warder Woreda of Dolo Zone and 47 suspected DF cases in Dolo Ado Woreda of Liban Zone of the Somali Region which are areas that had an experience of past DF outbreaks in 2017 and 2018 [43]. Contributing factors to the outbreaks include the weakened nutritional status of the community due to prolonged drought, population displacement, poor household water handling, living with ill people and lack formal education [19, 41].

Similarly, few serological studies reported DENV infections from different localities of the country. From northwest Ethiopia, in Gonder referral hospital, 7.5% current (acute) and 13.0% past DENV infections [44] and in Metema and Humera, 19% current and 21% previous DENV infections were reported from acute febrile patients [22]. In southern Ethiopia, in the Borena zone of the Oromia region, 22.9% against anti-DENV IgG and 7.9% against IgM of DENV infection were reported [45]. From the confirmed DF infections, the responsible serotypes for the outbreaks were DENV1-3 serotypes [33, 42, 44]. Even though DENV infection would be transmitted all year round in Ethiopia, the risk of contracting it in the country is the most significant during and immediately after the rainy season, which runs from June to August [43]. The close contact with DF patients, non-uses of bed nets, and the presence of stagnant water around the village were identified as risk factors for contracting DF in Ethiopia [18].

### Biology of DENV

#### Etiologic taxonomy and transmissions of DENV

DENV is a positive sense single-stranded encapsulated RNA virus and consists of three structural proteins and functional seven none-structural (NS) proteins [3]. It belongs to the genus *flavivirus* of the family *Flaviviridae* [5]. The family *Flaviviridae* comprises various genera, including the genus *flavivirus* that contains viruses such as DENV, yellow fever virus (YFV), Japanese encephalitis virus (JEV), tick-borne encephalitis virus (TBEV), West Nile virus (WNV), and Zika virus (ZIKV), genus *Hepacivirus* that contains hepatitis C virus (HCV), genus *Pestivirus* and many others like the newly proposed genus consisting of G Barker Virus (GBV) isolates [46–48]. Although members of the family *Flaviviridae* share similarities in morphology, genome organization, and replication, each genus differs in antigenic and biological properties. The genus *flavivirus* has distinct characteristics including most of the viruses in the genus are arthropods (mosquito or tick) borne and share closely related genomic organization and sequence homology, leading to antigenic cross-reactivity among members [47].

DENV is a roughly spherical virus particle with a diameter of approximately 50 nm, and based on cross-reactivity assays, the virus consists of four independent antigen-related serotypes (DENV1-4) [5, 49]. Epidemiologically, like in genetic diversity, transmission dynamics, and epidemic potential, all serotypes are almost similar, except the DENV-4 serotype which is genetically quite distinct [50]. The DENV exhibits two distinct morphological forms, namely, the intracellular immature virion and mature virion forms. The mature virion is distinguished by two virus-encoded membrane-associated E and M proteins, forming a relatively smooth surface. However, the intracellular immature virion has E protein and a precursor membrane (prM) protein which will be cleaved proteolytically into the M protein during maturation. The immature virion spikes the surface in an asymmetric, upright manner by undergoing extensive rearrangement of intracellular virus-encoded surface proteins upon acidification during maturation in the infected cells [51, 52]. However, partially mature/immature forms may sometimes be released from infected cells [52]. All serotypes have similar geographic distributions and host/vector associations as well as all are known to cause the entire spectrum of DF diseases; considering reasons to be a single species even if they have quite distinct genetically and antigenically [53]. In addition to the four worldwide identified DENVs, a fifth serotype (DENV-5) has been detected from a suspected patient in Malaysia in 2007 using isolation and genetic sequence analysis and announced in 2013 [54].

The DENV serotypes can be undergone to several subtypes/ lineages or genotypes based on several changes in the viral genome [49, 55]. DENT-1 was previously classified into five (IV) genotypes based on the sequence of the E gene. Of these, genotypes I, IV, and V appeared to predominate, while genotypes II and III appeared to be dormant [49, 56]. DENV-1 was classified recently into three genotypes (I, II, and III) by whole-genome sequencing [57, 58]. However, the role of different genotypes in triggering outbreaks is poorly studied [58]. DENV-2 consists of 6 genotypes, including Asian/American, Asian I, Asian II, Cosmopolitan, American, and Sylvatic, and 15 other subpopulations/ lineages. These variations are occurred when few codons in envelope genes confer antigenicity and lineage diversity to American strains of the Asian/American genotypes, codons from NS genes of DENV-2 confer lineages diversity to the Asian I, Cosmopolitan, and Silvatic genotypes [59]. DENV-3 consists of five genotypes ((I, II, III, IV and V) of which genotype III formed three heterogeneous subpopulations (III-a, III-b, and III-c) [60, 61]. Subpopulations of genotypes I, II, and III-a include Asians, and III-c and IV includes Americans strains. The III-b subpopulation contains mainly American strains and a minority of South Asians, but many genotype III strains and genotype V strains contain Asians and Americans [61, 62]. DENV-4 consists of five genotypes (I, IIA, IIB, III, and Sylvatic) [60, 63]. Genotype I was found in the Philippines and genotype IIA is common in Southeast Asia and China. Genotype IIB has been isolated in Southeast Asia and the Pacific Islands, and genotype III has been reported only in Thailand [63, 64]. The genotype and serotype diversity are primarily due to the high rate of genetic mutation driven by error-prone RNA-dependent RNA polymerases; the RNA polymerase lacks proofreading activity and generates mutation approximately per round of DENV genome replication [49]. On the other hand, a serotype can provide lifelong immunity to the infected person only against that specific serotype [65, 66]. Hence, the diversities in the genotypes and serotypes are major challenges in the development of a tetravalent vaccine.

Transmissions of DENV infection to humans can be known to undergo two types of transmission cycles, called urban and enzootic cycles [49]. The urban transmission cycle to humans occurs from domestic/peri-domestic habitats by female mosquitoes mainly of the species *Ae. aegypti* and, to a lesser extent, *Ae. albopictus* [5, 59]. The mosquitoes are infected when they bite a person infected with the virus, consequently, the infected mosquitoes can, therefore, extend the virus to other people through bites. These mosquitoes prefer to bite people during both the day and night and live indoors and outdoors near people [67]. Unlike many *flavivirus* DENVs are confined to their natural vertebrate host range that use primates as their amplification and reservoir hosts. The DENV transmission that occurs as an enzootic cycle would be in non-human primates of sylvatic habitats and arboreal mosquitoes, such as *Ae. taylori* and *Ae. fucifer* [59]. The urban transmission cycles are an endemic or epidemic cycle that takes place between human reservoir hosts and the mosquitoes, where larval maturation occurs around domestic water containers. DENV cannot be spread directly from person to person; however, infected humans are known to carry the infection from one country to another or from one area to another during the stage when the virus circulates and reproduces in the blood system [5].

### Structural components of DENV

The three virus-encoded structural proteins of DENV are C protein (12 kDa), M protein (8 kDa) which is cleaved from PrM protein (21 kDa) and E protein (53 kDa), whereas the seven NS proteins are NS1, NS2A, NS2B, NS3, NS4A, NS4B, and NS5 [68]. The virion particle is organized as virus-encoded outer protein layer, host cell-derived lipid bilayer envelope membrane, and an inner spherical nucleocapsid core [69]. On the outer surfaces, 180 copies of each E and M/PrM protein are anchored into the lipid bilayer membrane and spanned through the membrane to form an icosahedral arrangement. In the mature DENV and *flavivirus*, the 180 copies of 395 residues of E monomer proteins are organized into 90 head-to-tail homodimers on the outer layer. Each E monomer protein is composed of three domains that are arranged as central domain I connecting immunoglobulin-like domain III to a dimerization domain II [70], a membrane-proximal stem, and a transmembrane anchor [71]. Domain III is supposed to interact with host cell receptors for entry and key epitope to bind neutralizing antibodies [55]. The membrane-proximal stem has two predicted amphipathic helices of hydrophilic and hydrophobic parts lie against the viral membrane and span to the length of domain II during fusion. At the tip of the domain II, there is a hydrophobic fusion loop which is exposed after the removal of the pr peptide from prM and is important for interaction and fusion with host membranes [71].

The prM protein is cleaved at position 91 by furin or furin-like protease to produce the pr peptide and M protein. The M protein contains an N-terminal loop (the first 20 residues), α-helical domain (MH), and two transmembrane spans (MT1 and MT2). The MH domain which is a highly conserved residue located 20-38aa downstream from the prM cleavage site can regulate prM cleavage during viral particle maturation and host cell entry [72]. The cleavage of the ‘pr’ peptide from prM is occurred to keep the M protein remains transmembrane under the protein E shell in the mature particle during maturation [68]. The lipid bilayer envelope membrane, which encloses the central spherical core of the virion, is taken from the host endoplasmic reticulum (ER) membrane during the maturation process [73].

**Fig. 1:**
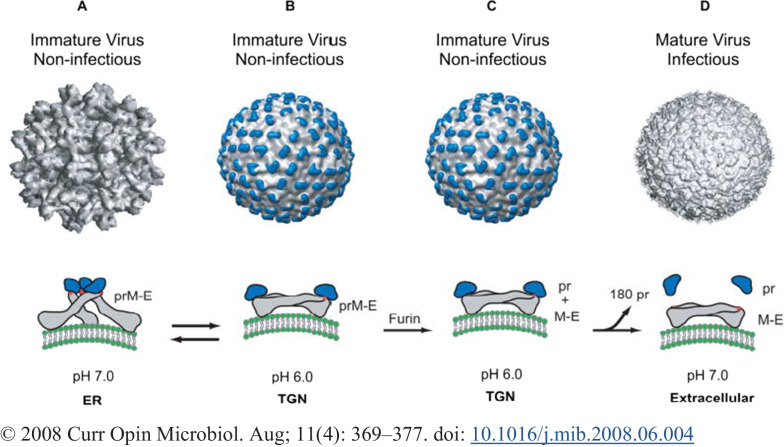
Structure of the dengue virion and conformations of the E protein

**Fig. 2:**
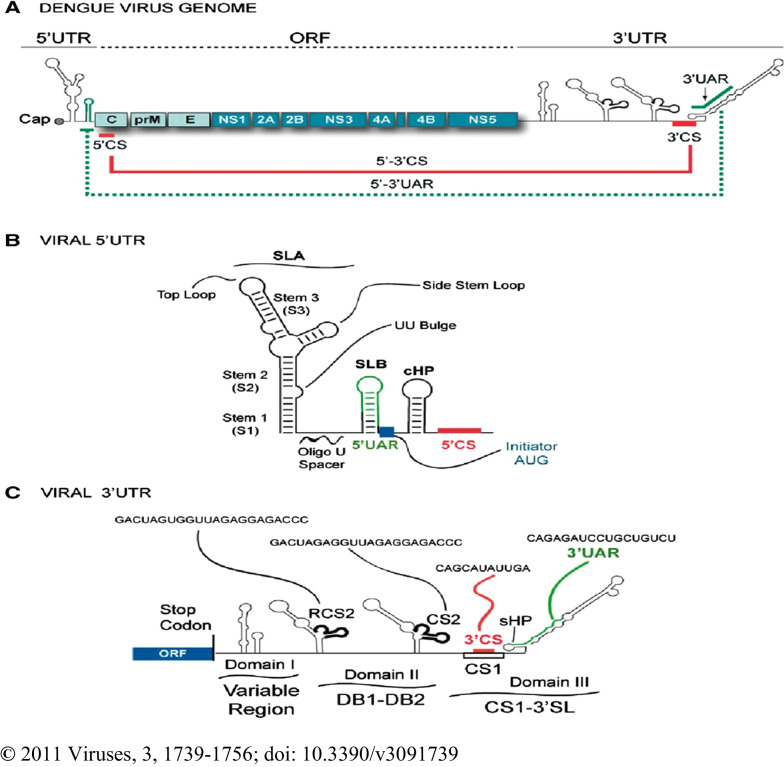
Schematic representation of the DENV genome: **A** ORFs showing structural proteins (C-prM-E) and NS proteins (NS1–NS2AB–NS3–NS4AB–NS5) and 5′ and 3′ UTRs. Positions of complementary sequences are indicated by solid lines for 5′–3′CS and dashed lines for 5′–3′UAR. **B** Predicted secondary structure of the 5′ UTR of the genome. Structural elements of the 5′ terminal region include stem loop A (SLA), stem loop B (SLB), oligo (U) track spacer, translation initiator AUG, capsid region hairpin (cHP), and 5′ CS element. **C** Predicted representation of RNA elements at the 3′UTR of the genome. Predicted secondary structures of three defined domains are shown: domain I (variable region, VR), domain II (dumbbell structure, DB1 and DB2), and domain III (conserved sequences CS1 and 3′SL). In addition, the respective positions and sequences of the conserved elements corresponding to RCS2, CS2, 3′CS and 3′UAR are indicated [76]

During reconstructions by cryoelectron microscopy and fitting of the known structure of E glycoprotein, the viral surface envelope exhibits an icosahedral scaffold of 90 E glycoprotein dimers [69]. The conformational changes take place on the two surface proteins by different environmental levels of pH, confer on the surfaces unique structural features of the immature and mature DENV forms [74]. In the immature form, the PrM and E form 90 heterodimers that extend as 60 trimeric spikes on the surface of the particle (Fig. 1A), wherein the mature particles the E protein form 90 homodimers of 30 raft-shaped groups of three E protein dimers that lie flat against the viral surface to form a ‘smooth’ herringbone-like pattern (Fig. 2D). During transition through the trans-Golgi network (TGN), predominantly conformational changes happened in the E protein due to pH level change determines to occur the structural transition from spiky immature to smooth shape mature [73]. Using the knowledge of E protein mass and comparing the size with other viruses, it was suggested that DENV may have three E subunits per icosahedral asymmetric unit [69]. The three E-monomers present in each icosahedral asymmetric unit indicate that the DENV virion lacks true T = 3 symmetry; this may contribute to its presence in three chemically distinct environments and may play different roles when present in different stages of the disease [66].

The central core of the virus is nucleocapsid, consisting the RNA genome molecule merged with C protein to form a central spherical core [66]. The NS proteins are functional proteins for a sequence motif characteristic, including viral serine protease, RNA helicase, and RNA-dependent RNA polymerase. They involve in controlling, coordinating and regulating various intracellular processes of viral life cycles [47, 75].

### Genome of DENV

The DENV comprises of about 11 kb long single-stranded positive-sense RNA molecule genome. The genome has single open reading frame (ORF) flanked by 5′- and 3′-terminal untranslated regions (UTRs) (Fig. 2A). The ORF encoded for a single of about 3390 residues of large polyproteins [53]. The 5′ and 3′ UTRs are non-coding regions used as RNA genome maintenance. The UTRs containing conserved sequences are cis-acting elements that regulate the processes of genome amplification, translation, and packaging by altering stability, localization, and translational efficiency [25, 50, 55, 76].

The 5′ UTR is an upstream region of ORF which is a short and highly structured sequences region of between 95 and 101 nucleotides of the four serotypes. The region has two structural domains of distinct functions during RNA synthesis. The domains are separated by a short oligo(U) sequence that functions as a spacer to enhance viral RNA synthesis. The first domain is about 70 nucleotides sequence, predicted to fold to large stem-loop A (SLA) which is proposed to act as a promoter for the viral RNA-dependent RNA polymerase (NS5). SLA shows portions of three helical structure regions (S1, S2, and S3), a side stem-loop (SSL), and a top loop (TL) which are essential structures recognized by viral RNA-dependent RNA polymerase (NS5) during viral RNA synthesis. The S1 and S2 regions represent one of the most conserved elements, but the sequence and structure of the S3 and side stem-loop show the most variation in *flaviviruses*. The second domain of 5′ UTR is a 16-nucleotide-long sequence which is predicted to form a short stem-loop B (SLB). The SLB is identified as a 5′ upstream AUG region (5′UAR), which is a complementary region to the counterpart 3′UAR present at the 3′ ends of the genome. The downstream of 5′ UTR is positioned by ~ 100 nucleotides long coding sequence for C that contains a highly conserved in all four DENV serotypes 5′ complementary sequences (5′CS) to 3′CS, a stable capsid region hairpin (cHP) and RNA element that modulates DENV replication in mosquito and mammalian cells. The 5′ CS, which is an 11 nucleotides long (134-UCAAUAUGCUG), mediates long-range RNA–RNA interactions between the ends of the RNA for the genome cyclization. The cyclization of the viral RNA occurs when the 5′UAR and 5′CS hybridize with the counterparts in the 3′ UTR, which is a process required for transferring the viral polymerase from the 5′ SLA to the 3′ ends to initiate genome replication (Fig. 2B) [53, 76–81].

The 3′UTR is a relatively long nucleotide sequence region that comprises about 470, 450, 430, and 385 (shortest) nucleotides sequence for DENV-1, -2, -3 and -4, respectively. The 3`UTR lacks a poly (A) tail (polyadenylation), which is a crucial tail for stimulation and stabilization of cellular mRNAs translational initiation but ends with a conserved 3′ stem-loop (3′SL). The 3′ UTRs of DENV and ZIKV have 3 major domains. Domain I: A stem-loop (SL) domain that immediately follows the stop codon of NS5 and a highly variable region inside the 3′ UTR. In the DENV serotypes, SL has a significant sequence and length variation that may vary from less than 50 nucleotides to greater than 120 nucleotides between the serotypes. Domain II: A dumbbell (DB) characteristic shape and duplicate in tandem. This domain has conserved CS2 and its repeated CS2 (RCS2) sequences. Domain III: The most conserved region of the 3′UTR, containing a CS1 element which is followed by a terminal 3′SL structure. CS1 is a structure involved in long-range RNA–RNA interaction between the end of the viral genome during cyclization (Fig. 2C) [53, 76, 81].

### Life cycle of DENV

**Fig. 3:**
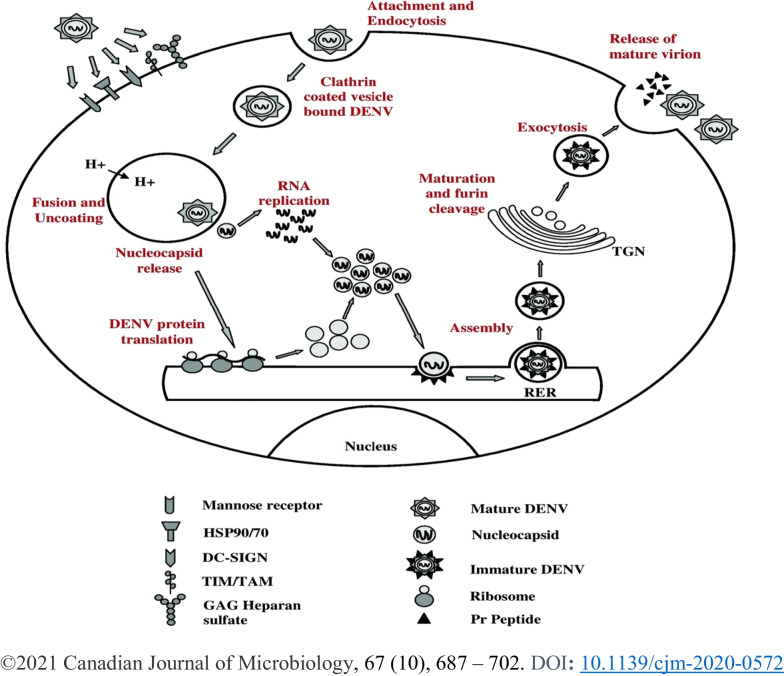
Step-by-step processes of dengue virus entry in the host cell and its life cycle

DENV infection and replication require step-by-step processes in host immune cells using cellular machinery. The DENV targets immune cells, including dendrite cells (DC), skin Langerhans cell, B cells, T cells, monocytes, macrophages, lymphocytes and liver cells to infect [82, 83]. The life cycle of the infection occurs in a subsequent fashion, as detailed below and depicted in Fig. 3.

### Attachment and cell entry

DENV attaches to the surface of the immune cells and enters the cells by a process known as endocytosis. During the binding to the immune cells, receptors on the surface of the cells trigger the virus to be taken in. The cognate receptor molecules such as glycosaminoglycans (GAG), lipopolysaccharide-binding protein in association with CD14 molecules, heparan sulfate and lectin-like receptors such as dendritic cell-specific intercellular adhesion molecule 3-grabbing non-integrin (DC-SIGN) are crucial for DENV infection. FC receptor involves in the mechanism referred to as antibody-dependent enhancement (ADE) dengue infection. During infection, E protein binds to the relevant receptors and triggers receptor-mediated endocytosis via clathrin-coated vesicles, sac-like structures, called endosomes [55, 84, 85]. DENV lands on the cell surface and either rolls over various surface receptors or migrates diffusively as a virus-receptor complex toward pre-existing clathrin-coated cavities during the process [86]. Alternatively, the entry of DENV into target cells can also occur independent of clathrin, caveolae and lipid rafts, depending on a non-classical endocytic pathway, for example, via dynamin [87].

After internalization, the virus particles are delivered to Rab5-positive early endosomes and mature into Rab7-positive late endosomes, where membrane fusion exclusively occurs [86]. The endosome’s interior pH can be decreased by proton pumps which trigger the virus to change the protein E conformation to form spike-like structures. As a postulate, the acidic pH in the endosomes triggers E homodimers dissociation, leading domain II to project outward and exposing the hydrophobic fusion loop peptide to the target endosome membrane [73]. The hydrophobic residues of the fusion loop penetrate the membrane of the endosome. Domain III also shifts and folds back toward the fusion loop peptide into a hairpin-like conformation [88]. Subsequently, the membrane-proximal stems span the length of domain II. Finally, the stems are aligned across the entire length of domain II to bring the TM anchor and the fusion loop together, completing membrane merger and pore formation (Fig. 3). The pore helps the virus to release the nucleocapsid into the cytoplasm [66, 89].

### RNA uncoating and translation

The viral translation and replication to be undertaken, the nucleocapsid needs to be broken and leave the C protein aside to release the viral RNA into the cytoplasm. The C protein is a highly basic protein that binds to viral RNA with high affinity but low specificity. Uncoating of the C protein from the viral genome occurs by an unexplained mechanism. The non-degradative steps of ubiquitination, however, are thought to function in genome uncoating [90]. In support of this thought, some studies suggested that inhibition of ubiquitination blocks the uncoating of the DENV genome, by demonstrating that the inhibition of the ubiquitin E1 activating enzyme stabilizes the viral genome by retaining it in endosomes or nucleocapsids during infection [2, 91]. The uncoated viral genome translocation to the rough endoplasmic reticulum (ER) occurs through still exactly unknown mechanisms, though believed that constant alteration of cytoskeletal machinery facilitates the translocation [92].

Translation of the genome RNA occurs at the surface of the ER membrane to produce viral proteins, subsequently initiating the critical step of genome RNA synthesis and amplification [93]. The members of the *Flavivirus* genome translation initiation are canonical cap-dependent. The genome RNA contains, like cellular messenger RNAs (mRNA), an m7GpppN-cap structure at the 5′ end though unlikely to lack a 3′ poly-A tail [81, 94]. To mediate genomic mRNA cyclization that is occurred to stimulate and stabilize translation initiation, the *Flavivirus* genome does not have a poly (A) tail binding to a poly (A) binding protein (PABP) to interact with the cap-bind eukaryotic translation initiation factor 4 complex (eIF4F). However, the eIF4F which is a crucial regulator of the cellular translation initiation complex for the cap-dependent translation-like DENV recognizes and binds to the m^7^GpppN cap structure at the 5′ end of mRNA and bridges it to the 40S ribosomal subunit to translate into a single polyprotein [95]. DENV also has relied alternatively on cap-independent cellular translation initiation to enable viral protein synthesis [95]. During the inhibition of cap-dependent translation by targeting the cap-binding protein eIF4E, the DENV replication and translation are unaffected [96]. However, the internal ribosome entry site (IRES), a region in the mRNAs that allow the internal initiation of translation, has not been identified for the *Flavivirus* [97]. Therefore, a study suggested that cap-independent translation appears to be regulated by both 5′ and 3′UTRs [93]. The ribosomal subunit and associated factors would recruit viral mRNA and scan 5′UTR until getting the AUG starts codon. The AUG selection may be assisted by a secondary structure element called cHP, located at 14 nucleotides downstream of the start codon and stops the 40S ribosomal subunit to ensure correct start codon selection [98].

During the polyprotein translation, signaling and stopping translates sequence direct back and forth translocation across the ER membrane and co- and post-translationally cleaved the polyprotein by viral and cellular proteases to produce the viral proteins. Specifically, cleavage of the polyprotein at conserved sites by the viral serine protease (NS2B/NS3), or by a host-derived signalase to cleave pr/M by furin or furin-like protease after assembly [47]. The three structural proteins occupy the N-terminal of the polyprotein and are arranged in the order of highly basic C protein (100 amino acids), followed by the prM protein (166aa) which proteolysis into M protein (75aa) during maturation, and then E protein (495aa) [47, 75]. In the meantime, the *Flavivirus* manipulate host cell gene expression at the translational level to facilitate the production of viral proteins and create a replication-friendly cellular state [93].

### RNA replication

The Flaviviridae family viruses have an identical structural arrangement and positive sense single-stranded RNA genome. The viral genome is an mRNA template for the replication of viruses into complete intermediate negative-sense single-stranded RNA upon entry into the host cell [47]. The positive-sense RNA viruses possesses a limited number of viral replication proteins, consisting of 3–10 genes; however, they extensively use host proteins, membranes, lipids, and metabolites during the life cycle process, including replication [99]. The positive sense RNA viruses induce massive rearrangements of intracellular membranes to create ultra-structural microenvironments derived either from the ER, mitochondria, Golgi apparatus, plasma membranes, or other organelles for virus RNA replication. These microenvironments are organelle-like membranous structures that favour replication by coordinating various steps of the viral life cycle through spatial separation of the replicating RNA from the ribosome and assembly to the C protein. Moreover, besides increasing the concentration of components required for efficient replication and assembly by reducing the diffusion space, these organelle-like structures also protect viral RNA from cellular nucleases and innate immunity-triggering pattern recognition receptors [100]. The *flavivirus-*induced membraneous microenvironment compartment of the RNA replicate is a vesicle packet (VP), created by ER invagination. The VPs are forms of a continuous membranous network in the ER, where functional viral replication complexes (VRCs) are assembled and to create isolated compartments to be hidden from the viral double-stranded RNA (dsRNA)-activated innate cellular immunity [101]. In the VPs, the VRCs function like a molecular factory by coordinating viral RNA replication through accommodating most of the viral NS proteins, such as NS3, NS4B, and NS5, viral replication intermediate dsRNA, and the host factors [93, 102–104].

The DENV RNA serves as a template for replication and actively provides regulated signals that act as promoters, enhancers, and silencers of RNA replication. The elements of the regulate signals for RNA replications are located within the 5′ and 3′ UTRs and in the viral coding sequences [76]. The RNA-dependent RNA polymerase of the NS5 protein initiates the viral RNA synthesis by binding to SLA located in the 5′ UTR of the viral RNA [105]. In *flavivirus*, the presence of the complementary sequence 5′–3′ CS and the cyclization sequence 5′–3′ UAR between the 5′ and 3′ base pairs are essential for viral RNA synthesis. To reach the replication initiation sites at the 3′ ends of the RNA molecule, the SLA-bound polymerase exploits the long-range 5′–3′ RNA–RNA interactions within the template mediated by genome 5′–3′CS and 5′–3′UAR hybridizations of the long RNA molecules [76]. The change in a hybridization of complementary sequences leading to RNA cyclization exposes the 3′ end of the viral genome, which is a template during the initiation of negative-sense RNA synthesis [77, 78, 106]. The RNA-dependent RNA polymerases, along with the viral protease/helicase NS3, other viral NS proteins, and presumably host factors, catalyze the enzymatic reaction process to synthesize a negative sense RNA that will serve as a template for the amplification of positive sense genomic RNA [107]. The progeny viral RNA synthesis occurs by asymmetric, semi-conservative replication on the replicative form (RF) templates or dsRNA recycling [108], because the viral replications are the production of 10–100-fold more positive-sense progeny RNA than intermediate negative-sense RNA. As the viral RNA was required to be recruited to the replication compartment for replication, the newly synthesized positive-strand RNA is required to be released from the compartment after the optimum RNA is synthesized.

### Virion assemblage and liberation

Virion assembly involves encapsulation, envelopment and acquisition of a lipid envelope containing glycoproteins by budding across the intracellular membrane. The NS2A protein recruits viral genome RNA, structural proteins and proteases to virion assembly sites and orchestrates nucleocapsid and virus formation. The 3′UTR end of the viral genome that serves as a recruitment signal for packaging is linked to the cytoplasmic loop of NS2A, allowing NS2A to recruit nascent RNA from the VRC to the virion assembly site. NS2A also recruits the C-prM-E polyprotein and the NS2B–NS3 protease to the virion assembly site through interactions with prM, E, and NS3, resulting in coordinated C-prM-E cleavage [109].

In members of the genus *Flavivirus*, nascent RNA is assembled into virions by encapsulation, after which the envelope buds into the ER lumen. Primarily mature C proteins are encapsulated into the viral RNA to form nucleocapsids, which are subsequently loaded with prM and E proteins and conquer a lipid bilayer envelope containing glycoproteins by budding across the intracellular membrane to form virions. In DENV VPs, the opening into the cytosol allows access for metabolites [such as nucleoside triphosphates (NTPs)] to the VRC and for newly replicated viral RNA to exit the VP for translation or assembly of virus particles. The replicated DENV genomes released through the VP pore can be used directly for packaging into virion particles and buds through the ER membrane in near the VP [100]. The close proximity of the replication and assembly sites to the VP may reveal DENV RNA selectivity for encapsulation by shifting the balance from RNA translation to genome encapsulation. This transient regulation is C protein accumulates to sequester viral RNA for replication DENV life cycle, whereas released viral RNA is preferably used for translation during early timepoints after infection when low levels of structural proteins are present [100]. The formed virus particles are transported to cytoplasmic vesicles via the secretory pathway before being released by exocytosis (Fig. 3). The initial immature virion, containing 60 prM and E heterotrimers in an icosahedral arrangement on the surface virion, migrates through the Golgi network, where the acidic environment triggers cleavage of prM by the cellular furin protease, leading to infectious mature virions production [110, 111].

### Pathogenesis of DENV infection

Pathogenesis of DENV infection is complex and not fully understood though the spectrum of the pathogen severity of all serotypes ranges from mild DF to severe dengue hemorrhagic fever (DHF) and dengue shock syndrome (DSS) [112]. The pathogenesis is attributed to a complex interaction of the virus, host genes, and immune responses of the host [113]. Because, the clinical features and severity of DF occur during the existence of factors like being a neonate or young child, female, high body mass index, genetic polymorphisms and previous infection with DENV-1 if the patient contracts DENV-2 or DENV-3, co-morbidities, such as diabetes and asthma disease [9, 25]. During severe DF cases, the DENV induces blood coagulation abnormality and plasma leakage and increases vascular fragility to lead to DHF. Furthermore, the virus increases capillary permeability to cause a body fluid loss that results in a hypovolemic shock DSS and multiple organ failures [114]. Hence, the patho-physiological features of severe DF may be due to plasma leakage and abnormal hemostasis. The plasma loss in DF and its complication outcome have been known for the past 10 years, but the mechanism of the expression demonstrated by the virus remains unclear [115]. On the other hand, the DENV infection severity peaks after the virus has been cleared by the host immune system, not during the viral load is at peak [116]. It is an important finding to confirm that the host immune response plays a crucial role in the pathogenesis of DENV infection.

**Fig. 4:**
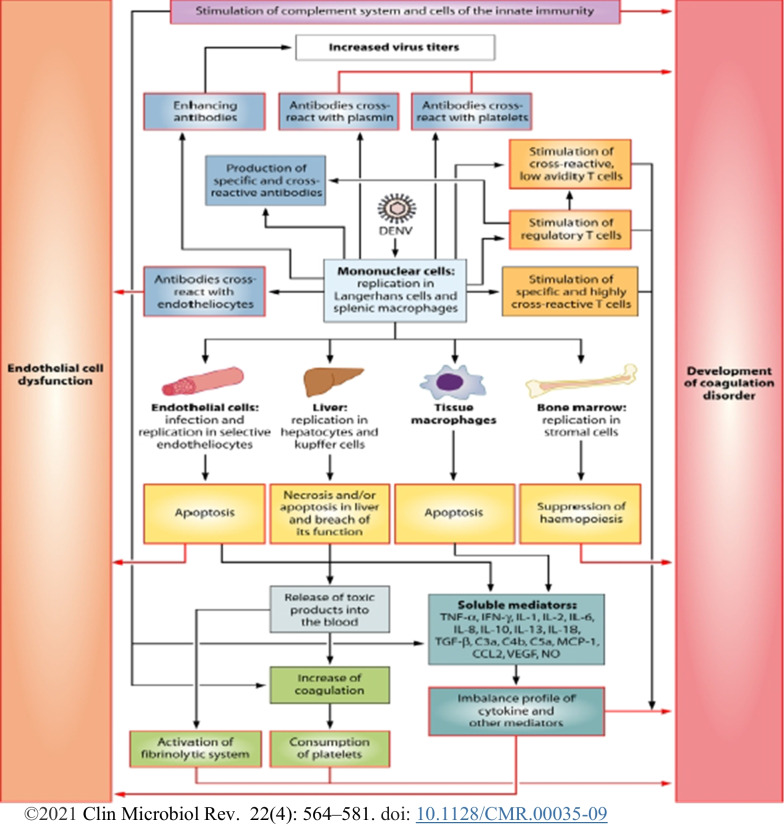
Model for the pathogenesis of DF, DHF, and DSS. Black arrows—processes leading to the indicated event; colored boxes with white centers–pathological events. Each event will ultimately affect the EC or the hemostatic system (purple arrows)

The host organs and tissues’ tropism of DENV are considered major determinants in the pathogenesis; however, the absence of adequate tropism assays and animal models has hampered the understanding of replication tropism in the tissues cell for pathogenesis [117]. The presence of DENV (−) sense RNA or NS3/NS5 proteins in specific cells of tissue may indicate DENV replication, as these antigens are present when DENV replicates, whereas detection of other DENV antigens (E, prM, C, (+)-sense RNA) may indicate no active replication of DENV in cells, as they do not permit replication of DENV rather cells may non-specifically take up viral RNA and other antigens from the surroundings [112]. In vitro and autopsy studies, after mosquito bites, cells of the skin are infected and deliver DENV to the draining lymph nodes through lymph in which resident macrophages and different unknown cells are infected and deliver the virus to the lymphatic and vascular system, leading to the infection of bone marrow and spleen [112]. Then, Peyer’s patches and lymph nodes are infected or may acquire DENV immediately from draining lymph nodes or through bone marrow and spleen and numerous non-lymphoid organs such as stomach, thymus, lung, brain, gastrointestinal tract, liver, kidney, and heart are likely infected [112]. It is clear that the immune system cells and endothelial cell (EC) lining of blood vessels play a vital role for DENV tropism and severe pathogenesis. The infection of the DENV to host cells such as macrophages, hepatocytes, and EC influences the hemostatic and immune responses to the virus, representing a considerable risk factor for severe illness development. Infected cells die in particular via apoptosis and, to a lesser extent via necrosis; necrosis releases toxic products, which activate coagulation and fibrinolytic systems. On the other hand, the hemopoiesis process is depressed, resulting in to decrease in thrombogenicity in the blood depending on the extension of the infection to the bone marrow stromal cells and levels of IL6, IL8, IL10, and IL18. The high viral load blood, viral tropism for the EC and platelet dysfunction by severe thrombocytopenia result in a high capillary fragility to cause DHF and clinically manifested as petechiae, easy bruising, and gastrointestinal mucosal bleeding [118]. The DENV infection stimulates the development of specific antibodies and cellular immune responses despite the immune response aggravating the pathogenesis. Studies identified that when IgM antibodies produced against DENV can cross-react with EC, platelets, and plasmin to result in a cycle of amplification for higher vascular permeability and coagulopathy, the improved IgG antibodies bind heterologous viruses during secondary infection of different serotypes and improved antigen-presenting cells (APCs) infection to contribute to the highest viral load viremia in some patients during secondary infection. The viral load overestimates both low and high avidity cross-reactive T cells as in some haplotypes of HLA; cross-reactive T cells delay the virus clearance, producing high levels of pro-inflammatory cytokines and other mediators. The high levels of soluble factors induce changes in the CE leading to coagulopathy and attributing plasma loss to DSS (Fig. 4).

Most primary infections usually present as an asymptomatic or mild febrile illness but also cause hemorrhagic fever in some patients, particularly babies born to a DENV-immune mother. Subsequent infection with different serotypes can lead to severe clinical manifestations, such as DHF and DSS [119, 120]. The DENV-infected person can produce lifelong immunity against only the infecting serotype; however, the product provides only temporary and partial immune responses against other serotypes [66]. The severity of DENV increases during secondary infection of different serotypes due to the weakly neutralizing antibodies from the first infection binding to the second serotype and enhancing antibody-dependent enhancement (ADE) infection by Fc-receptor-bearing immune cells, such as monocytes and macrophages [85]. The cross-reactivity of the primary DENV infection antibodies forms a combination with a second infecting serotype to form infectious immune complexes that enter Fc-receptor-bearing cells, ensuing an extended number of infected cells and increased viral output in line with the infected cell [119]. The synergistic actions of viral serotypes and various host factors, including ADE, memory cross-reactive T cells, anti-DENV NS1 antibodies, and autoimmunity play a vital role in the severe manifestations of DF in humans [113]. The severities of DF are likely to be multi-factorial, though the mechanisms leading to severe are yet to be understood.

### Clinical manifestations of DENV infection

DENV and other *flavivirus* can cause serious diseases ranging from febrile illness to fatal hemorrhagic, neurologic, and gastrointestinal symptoms [121]. DENV infections, following an incubation period of 4–10 days after being bitten by an infected mosquito, may have clinical features that last 2–7 days including asymptomatic or may lead to undifferentiated fever, DF or DHF with plasma leakage that may lead to hypovolaemic shock, DSS [9]. Symptomatic DENV infections were classified into DF, DHF and DSS according to the 1997 WHO classification guidelines until 2009 [8]. However, in the 2000s, the DF expert groups agreed that DF is a fundamental disease that has different clinical manifestations and often has unpredictable clinical features and outcomes; DF cases reclassification into severity levels has strong potential for practical utility in clinicians’ decisions about, where and how intensively a patient should be monitored and treated [9]. Furthermore, the 1997 classification of DHF and DSS case definitions was too difficult to apply in resource-limited settings, and too specific and failed to identify significant proportion of severe DF cases, including cases of hepatic failure and encephalitis [122]. Consequently, in 2009 WHO categorized DF as non-severe and severe DF based on a set of clinical and/or laboratory parameters [9].

**Fig. 5:**
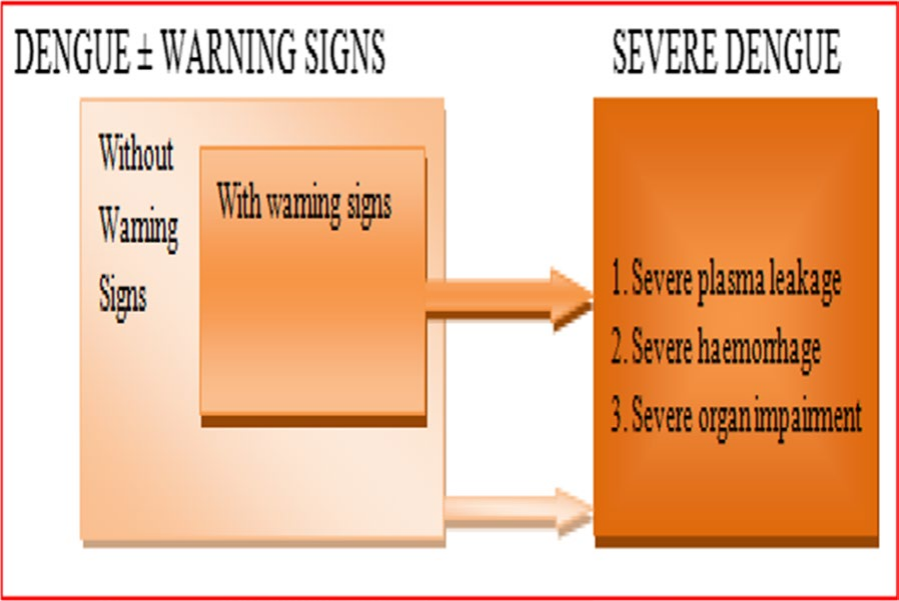
Suggested dengue case classification and levels of severity

Furthermore, the 2009 classification had split a large non-severe DF patient group for practical purposes into two categories: DF without warning signs (D − W) and DF with warning signs (D + W). Criteria for probable DF (D − W) would be living in or travelling to DF endemic area and showing fever and two of the following clinical symptoms: nausea (vomiting), skin rash, soreness, positive tourniquet test, leucopenia or any of the warning signs. The D + W patients, which require strict observation and medical intervention, can show all clinical symptoms of D-W, and abdominal pain or tenderness, persistent vomiting, clinical fluid retention, mucosal bleeding, lethargy/restlessness, liver enlargement > 2 cm, and/or laboratory findings with an increase in hematocrit (HCT) associated with a rapid decrease in platelet count. The criteria for DHF are all symptoms of DF and associated with hemorrhagic manifestations (positive tourniquet test or spontaneous bleeding), thrombocytopenia, and signs of increased vascular permeability that increase hemoconcentration or fluid effusion in the chest or abdominal cavity (Fig. 5). As the DHF severity extends, plasma leakage occurred to leads DSS and fluid accumulation with respiratory distress, severe bleeding based on clinician assessment, or severe organ involvement including liver (AST or ALT ≥ 1000), CNS (impaired consciousness), heart and other organs [9]. However, the 2009 WHO classification guidelines have been criticized for being too comprehensive, allowing several different methods of diagnostic criteria for severe DF and using non-specific warning signs as diagnostic criteria for DF. Besides, the guidelines have failed to have defined clinical criteria for the diagnosis of severe DF apart from providing laboratory breakpoints for transaminase levels, and determination of severity is subject to individual clinical judgment [122]. The 2009 WHO scheme is effective in identifying severe cases [123], but when the 1997 guidelines were followed, patients tended to fall into lower severity grades [124]. The 1997 classification appeared to identify truly severe cases, whereas the 2009 guidelines were more useful in recognizing a wide range of severe clinical manifestations.

The clinical features of DF frequently depend on the age of the patient. Infants and young children may have an undifferentiated febrile disease, often with a maculopapular rash. Older children and adults may have mild febrile symptoms or classic debilitating disease with rapid onset of fever, severe headache, retro-orbital pain, myalgia, arthralgia, and gastrointestinal discomfort, often with a skin rash and sometimes minor bleeding in the form of petechiae, nosebleeds, gastrointestinal bleeding and bleeding gums. In addition, in those with signs of bleeding, usually leucopenia and occasionally thrombocytopenia can be observed in DF [8, 125, 126]. In Children, DHF commonly presents with a sudden temperature rise accompanied by facial flush and other non-specific constitutional symptoms resembling DF, such as anorexia, vomiting, headache, and muscle or bone and joint pain. DSS is a shock and deterioration occurs suddenly after a fever of 2–7 days or shortly after defervescence (during the return of fever to normal), whereas DSS is a rapid, weak pulse (≤ 20 mmHg) or hypotension accompanied by cold skin and dizziness in the early stages of shock. Hence, for patients who do not receive prompt and appropriate treatment, a period of profound shock can occur, in which pulse and blood pressure are undetectable, leading to death within 12–36 h after the onset of shock [8, 9].

### Diagnosis and control of DENV infection

DF should be considered in a patient typically present with acute onset of fever, headache, body aches and sometimes rash spreading from the trunk and who lives in or recently traveled to a disease-endemic area in the 2 weeks before symptom onset [122]. The diagnosis can be performed by detecting the virus, viral nucleic acids, antigens, anti-DENV antibodies, or combinations of these techniques [5]. In the early stages of the disease (seven or less than 7 days after onset of illness), DENV infection can be diagnosed from serum, plasma, circulating blood cells, or from other tissues by detecting viral RNA with nucleic acids amplification tests, NS1 protein using some commercial tests and viral isolation in mammalian or mosquito cell culture to further genotyping and lineage for virus characterization [122]. DENV is thermally labile; RNA detection and isolation of the virus are highly dependent on well-preserved specimens for accurate diagnosis results [127]. The samples awaiting shipment to the laboratory should be stored in a refrigerator or freezer. That is, for storage up to 24 h, samples should be stored at 4–8 °C and for long-term storage samples should be frozen in a − 70 °C refrigerator or liquid nitrogen container [9].

For patients with suspected DF disease, serum specimens during acute phases (≤ 7 days after onset of illness) would be collected and diagnosed by detecting the viral RNA sequence by reverse transcription–polymerase chain reaction (RT–PCR) or NS1 protein and/or anti-DENV antibodies by enzyme-linked immunosorbent assays (ELISA) or rapid point-of-care tests [128]. IgM antibody capture enzyme-linked immunosorbent assay (MAC-ELISA) is used for the qualitative detection of DENV IgM antibodies starting 4–5 days after onset of symptoms and is also reliably detectable for approximately 12 weeks. The MAC-ELISA is based on capturing human IgM antibodies on a micro-titer plate using anti-human–IgM antibody followed by the addition of DENV antigens derived from the envelope proteins of the four serotypes. Plaque Reduction Neutralization Tests (PRNTs) that detect specific neutralizing antibodies against DENV and other flavivirus are performed on IgM positive patients to determine the cause of infection or to rule out other flavivirus, such as ZIKV, YFV and, in some cases, to determine the infecting DENV serotypes [5, 122]. Moreover, the recent development of ELISA and dot blot assays targeting the E/M and NS1 antigens has demonstrated that high levels of these antigens in the form of immune complexes are detected in patients with both primary and secondary dengue infections up to 9 days after the onset of illness. The NS1 glycoprotein is produced by all *flavivirus*, secreted by mammalian cells, and generates a very strong humoral response, so that detection of NS1 allows early diagnosis of dengue virus infection, although serotypes are not differentiated [9]. The nested RT-PCR protocol was developed using universal dengue primers targeting the C/prM region of the genome for the initial reverse transcription and amplification step, followed by a nested PCR amplification for identification of the infecting serotype-specific qualitatively [129]. Moreover, the combination of the four serotype-specific oligonucleotide primers in a single reaction tube which utilizes one-step multiplex RT-PCR was an interesting alternative to the nested RT-PCR [130]. The advancement of RT-PCR into real-time (rRT-PCR) by incorporating dyes and probes (SYBR green and TaqMan) in a single step is capable of providing quantitative data [131]. The presence of the virus by rRT-PCR or NS1 antigen in a single diagnostic sample is considered laboratory-confirmed dengue in patients with compatible clinical and travel histories [122]. For patient illness of more than 4 days after the onset of fever, DF can be diagnosed by testing serum for IgM antibodies produced against DENV using MAC-ELISA, whereas for patients presenting within the first week after fever, testing for DENV should include detection of rRT-PCR or NS1 and IgM [122].

After the acute phase of infection has subsided or after 7 days of fever onset, detection of IgM antibodies is the preferred method of diagnosis using ELISA and hemagglutination inhibition (HI), although NS1 has been reported positive up to 12 days after fever onset [9, 67]. If the DENV infection occurred in a person who had no previous *flavivirus* infections or had not been vaccinated against *flavivirus,* such as ZIKV, YFV, JEV, and TBE, patients would develop a primary antibody response which slowly increases for a limited time long [128]. The IgM isotype is the primary emerging antibody and detection rate in serum is increased as follows: by days 3–5 after the disease onset, it is detected in 50% of patients, by day 5 detection increased to 80% of patients and by day 10–99% of patients [9]. During DENV infection occurs in a place, where other potentially cross-reactive *flavivirus* such as ZIKV, WNV, YFV, and JEV are not a risk, a single serum sample IgM test result strongly suggests a recent DENV infection and should be presumed confirmatory for DF [67]. The IgM level peaks at about 2 weeks after disease onset and declines to undetectable levels after 2–3 months. Contrarily, IgG started to be detected at low titers in serum, usually at the end of the first week of onset, and slowly increases thereafter and is detected after months and even years [9].

During secondary DENV infection or after vaccination or infection with a non-dengue *flavivirus*, the IgG isotype antibody titers rise rapidly; the predominant antibody isotype is detected in secondary infection with high levels in an acute phase and lasts 10 months and sometimes lifelong [128]. During the convalescent phase, IgM antibodies can be reliably detected but negative for viral RNA or NS1 test [122]. The IgM levels during early convalescence are significantly lower within secondary than primary infections and may be undetectable depending on the tests used. Hence, IgM detection is a reliable serological diagnostic test target in primary DENV infections [132]. IgM/IgG antibody ratios and HI tests are used to distinguish between primary and secondary dengue infections [133]. IgM and IgG anti-DENV antibodies detection are useful to confirm recent or past infection, because where IgM can be formed about 1 week after infection and reaches their peak 2–4 weeks after the onset of disease, the formation time of IgG level is longer than that of IgM but IgG will stay in the body for many years [5]. The presence of IgM indicates a recent infection; the presence of IgG indicates a previous DENV infection. Similarly, during the clinical course, the IgG/IgM ratio plays an important role in differentiating DENV infection. A ratio of 1.10 or higher is found the optimal cutoff point for differentiating secondary from primary DENV infection [134].

DENV can be isolated from serum, plasma, and peripheral blood mononuclear cells and obtained from tissue autopsies (e.g., liver, lung, lymph nodes, thymus, or bone marrow), although specimens for isolation should be collected early in the infection process and during the viremia stage (usually before the 5th day) [112]. Cell culture is a widely used method to isolate DENV as a golden standard for DENV infection diagnosis, using the mosquito cell lines C6/36 (cloned from *Ae*. *albopictus*) or AP61 (a cell line from *Ae. pseudoscutellaris*) and rarely and mammalian cell cultures, such as Vero, LLCMK2, and BHK21. Consequently, the viral RNA genome sequencing is performed for the genotyping of the serotypes and to characterize the molecular epidemiology of DENV infections [135, 136].

Rapid diagnostic tests (RDTs) for detecting NS1 protein antigen, IgM, IgG, and IgA antibodies have been developed by many commercial companies and are widely used due to their ease of use and rapid results. In many DENV endemic settings and areas with limited laboratory diagnostic resources, RDTs provide opportunities for point-of-care diagnosis, as well as secondary infection or convalescent timepoints after recent infections [137]. The IgM-based RDT format alone is not sensitive enough for acute DF diagnosis. On the other hand, the IgG-based RDT format is not recommended for diagnosing of acute DF, because IgG antibodies persist for lifelong and are more likely to be misdiagnosed as false-positive. The NS1 antigen-based RDTs are important part of modern point-of-care diagnostics, but are sensitive only in the early stages of infection and are not suitable for single use in epidemic settings, where late clinical manifestations may occur [138].

Careful medical detection and monitoring of patients with DF can significantly reduce mortality from severe dengue [31]. Currently, there is no specific treatment for DENV infection. Symptoms of muscle pain, fatigue, and fever can be relieved and reduced by treatment with acetaminophen or pain relievers, such as acetaminophen. Non-steroidal anti-inflammatory drugs (NSAIDs), such as ibuprofen and aspirin, are not recommended, because these anti-inflammatory drugs have a blood losing effect and blood anticoagulants that can worsen the prognosis of diseases with a risk of hemorrhage. For severe DF, medical care by doctors and nurses familiar with the effects and course of the disease can reduce mortality from more than 20% to less than 1% by maintaining patients' fluid volume, essential for the management of severe DF [5, 8, 9]. Currently, Dengvaxia^®^ is the only DENV vaccine approved and in use in the United States for children ages 9 to 16 with laboratory-confirmed evidence of previous DENV infection and living in areas, where DF is common [67]. Control of DF/DHF relies primarily on the use of insect repellents, wearing long sleeves and long trousers, and mosquito repellent inside and outside the home. According to the Global Vector Control Response (GVCR) noted, epidemiological surveillance with case detection and control, and entomological surveillance and control would be pillars to prevent and control DENV infections [139].

### Limitations of the review

This review covers the most relevant aspects of DF global epidemiology, transmission, etiology, morphology, life cycle, genome, pathogenesis, clinical features, and diagnosis. Although this review article discusses DENV infection in detail, it has limitations. Only 139 research articles and organizations' report documents are covered, and the detailed aspect of DENV prevention and treatment were barely covered due to the absence of proven curative drugs. Only the DENV scenario observed in Ethiopia was detailed; other countries were not emphasized in-depth due to the time and length limitations of the article. This article also lacks information on the detailed genome segment-based DENV diagnostic method. It also does not include information on failures and progress in developing preventive vaccines against DENV.

## Conclusion

DENV infection is a complex systemic disease with severe medical and economic consequences. To fully understand the impact on patients and the general public, assessing the full spectrum of the DF disease burden would be substantial. Considering Ethiopia is a representative developing country, it has been experiencing at least one DF epidemic every year since 2013, severely depleting the country's economy and health system. The high vector burden and rapid increase in seroprevalence of both current and past DENV infections in febrile individuals suggest that mosquito-borne DENV infection plays an important role in the spread of the disease. Globalization, population growth, and urbanization, lack of sanitation facilities, change in climate and environmental factors ineffective mosquito control, and increased DENV surveillance are factors behind the increase in DF worldwide. In addition, major risk factors for individuals to contracting DENV and developing DF include poor nutrition, persistent drought, population displacement, poor water handling, living with the ill, and lack of formal education. Appropriate precautions are recommended to minimize the risk of DENV infection.

Efforts are being made to expand surveillance coverage, but achieving the goal of eliminating DENV by 2030 will require ensuring prevention and control practices in all sectors of healthcare organizations. However, there is still a long way to go before this global health burden is reduced and DF is eliminated. In the meantime, human struggles to achieve this without preventive vaccines and efforts must be made to utilize effective preventive vaccines.

## Data Availability

Not applicable.

## Abbreviations

ADE: Antibody-dependent enhancement
APCs: Antigen presenting cells
CDC: Centres for Disease Control and Prevention
CHIKV: Chikungunya virus
cHP: Capsid region hairpin
CS: Complementary sequence
DC-SIGN: Dendritic cell-specific intercellular adhesion molecule 3-grabbing non-integrin
DENV: Dengue virus
DF: Dengue fever
DHF: Dengue hemorrhagic fever
dsRNA: Double-stranded RNA
DSS: Dengue shock syndrome
D-W: DF without warning signs
eIF4F: Eukaryotic translation initiation factor 4 complex
ELISA: Enzyme-linked immunosorbent assays
GAG: Glycosaminoglycans
GBV: G Barker virus
HCV: Hepatitis C virus
IRES: Internal ribosome entry site
JEV: Japanese encephalitis virus
MAC-ELISA: IgM antibody capture enzyme-linked immunosorbent assay
PABP: Poly (A) tail binding to a poly (A) binding protein
rRT–PCR: Real-time reverse transcription–polymerase chain reaction
RT–PCR: Reverse transcription–polymerase chain reaction
SLA: Large stem-loop A
SLB: Short stem-loop B
TBEV: Tick-borne encephalitis virus
TGN: Trans-Golgi network
UTR: Untranslated region
VP: Vesicle packet
NV: West Nile virus
WNV: West Nile virus
YFV: Yellow fever virus
